# Are Tobacco Prices in Japan Appropriate? An Old but Still Relevant Question

**DOI:** 10.2188/jea.JE20210416

**Published:** 2022-01-05

**Authors:** Masao Ichikawa, Takahiro Tabuchi

**Affiliations:** 1 Department of Global Public Health, Faculty of Medicine, University of Tsukuba, Ibaraki, Japan; 2 Cancer Control Center, Osaka International Cancer Institute, Osaka, Japan

**Keywords:** affordability, economics, price, taxation, tobacco

Tobacco prices in Japan have increased due to a gradual tax hike from 2018 to 2021. The total amount of the tax increase in the past 3 years was 3 yen per stick or 60 yen (about 0.5 United States dollar [USD]) per pack.^1^ When taxes on tobacco were increased 10 years ago, Japan Tobacco criticized the move, suggesting that the level of taxation on tobacco was already sufficient in Japan and that tobacco prices were not lower than in other countries when considering commodity prices.^2^ This begs the question of whether tobacco prices in Japan are appropriate relative to the wealth of the country.

For cross-national comparisons of tobacco prices, affordability should be considered.^3^ The same price (eg, 1 USD for one pack of cigarettes) may be considered cheap and affordable in higher-income countries but expensive and less affordable in lower-income countries. There are several measures of tobacco affordability.^3^ Among them, the “relative income price” is readily available for cross-national comparisons including Japan. This measure is defined as the proportion of gross domestic product (GDP) per capita required to purchase 100 packs of cigarettes, with a smaller proportion indicating higher affordability.

We obtained data on the relative income price from the World Health Organization, which compiles tobacco tax and price information from Member States every 2 years.^4^ As of September 2021, the latest available year was 2018. To identify the level of Japan’s tobacco affordability, we compared the relative income price (ie, tobacco affordability) across 38 countries belonging to the Organisation for Economic Co-operation and Development (OECD) and examined its relationship with GDP per capita to investigate whether tobacco affordability is proportional. Data on GDP per capita in 2018 were obtained from the OECD.^5^

**Figure 1.**
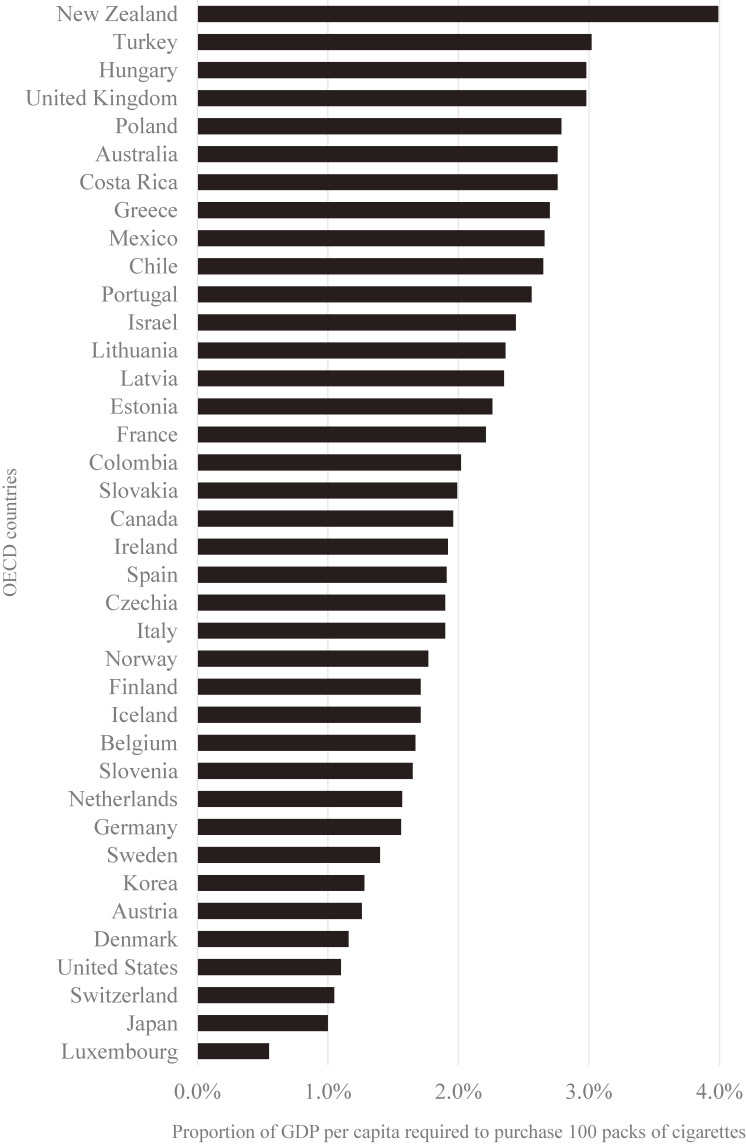
Proportion of GDP per capita required to purchase 100 packs of the most-sold brand of cigarettes among OECD countries in 2018. GDP, gross domestic product; OECD, Organisation for Economic Co-operation and Development.

The proportion of GDP per capita required to purchase 100 packs of the most-sold brand of cigarettes among OECD countries in 2018 is illustrated in Figure 1. Among the 38 countries studied, Japan was found to have the second highest affordability for tobacco, as the proportion was the second lowest, with 100 packs of cigarettes purchasable with 1% of GDP per capita. On the other hand, this figure was at least 2% (and even as high as 4%) in half of the OECD countries. To achieve this level of tobacco affordability, the tobacco price in Japan would need to be doubled.

**Figure 2.**
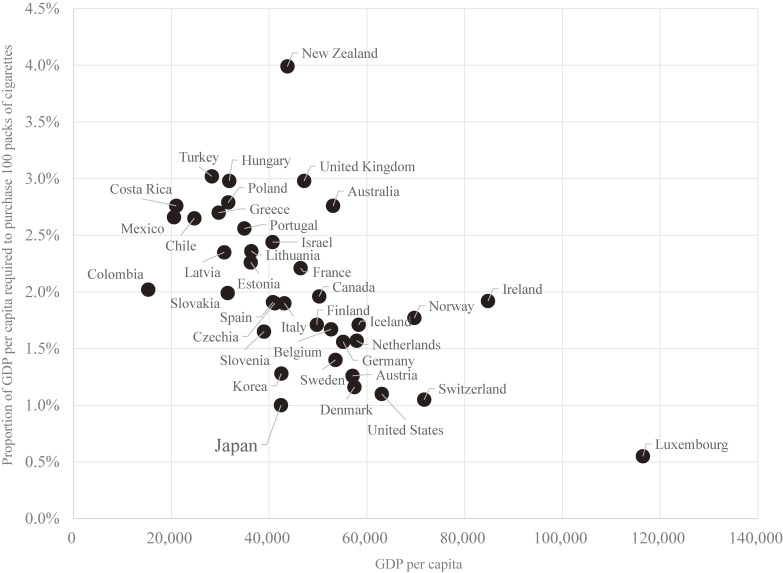
Relationship between GDP per capita and the proportion of GDP per capita required to purchase 100 packs of the most-sold brand of cigarettes among OECD countries in 2018. GDP, gross domestic product; OECD, Organisation for Economic Co-operation and Development.

Figure 2 shows the relationship between GDP per capita and the proportion of GDP per capita required to purchase 100 packs of cigarettes. The proportion tended to be lower in countries with higher GDP per capita, meaning that tobacco affordability tended to be higher with higher GDP per capita; however, tobacco affordability varied considerably among countries with a similar level of GDP per capita. This suggests that tobacco affordability is not necessarily determined by the level of GDP per capita but, instead, is impacted by other factors, such as tobacco policies inflating tobacco prices. In fact, tobacco affordability in all countries wealthier than Japan (except Luxemburg) was lower than that of Japan.

We acknowledge that tobacco affordability presented herein is based on the price of the most-sold brand cigarettes and GDP per capita, which reflects neither the income distribution in the population nor the income level in the smoking population. Tobacco affordability can be more accurately estimated with individual rather than aggregate data,^6^^,^^7^ but such estimates are not available for cross-national comparisons. In summary, tobacco prices in Japan appear to be low relative to the wealth of the country. The government should use tobacco price increases as an effective method of achieving a tobacco-free society.^8^^–^^10^
